# Bladder and bowel dysfunctions in 1748 children referred to pelvic physiotherapy: clinical characteristics and locomotor problems in primary, secondary, and tertiary healthcare settings

**DOI:** 10.1007/s00431-016-2824-5

**Published:** 2016-12-19

**Authors:** Marieke L. van Engelenburg–van Lonkhuyzen, Esther M.J. Bols, Marc A. Benninga, Wim A. Verwijs, Rob A. de Bie

**Affiliations:** 1 Department of Epidemiology, School for Public Health and Primary Care (CAPHRI), Maastricht University Medical Centre (MUMC+), PO Box 616, 6200 MD Maastricht, the Netherlands; 2 Department of Paediatric Gastroenterology, Emma Children’s Hospital/Academic Medical Center, Meibergdreef 9, 1105 AZ Amsterdam, the Netherlands; 3 Zuwe Hofpoort Ziekenhuis, Polanerbaan 2, 3447 GN Woerden, the Netherlands

**Keywords:** Constipation, Enuresis, Incontinence, Motor control, Pelvic floor muscles, Questionnaire

## Abstract

The aims of this study are to evaluate in a pragmatic cross-sectional study, the clinical characteristics of childhood bladder and/or bowel dysfunctions (CBBD) and locomotor problems in the primary through tertiary health care setting. It was hypothesized that problems would increase, going from primary to tertiary healthcare. Data were retrieved from patient-records of children (1–16 years) presenting with CBBD and visiting pelvic physiotherapists. Prevalence’s of dysfunctions were compared between healthcare settings and gender using ANOVA and chi-square test. Agreement between physicians’ diagnoses and parent-reported symptoms was evaluated (Cohen’s Kappa). One thousand seventy hundred forty-eight children (mean age 7.7 years [SD 2.9], 48.9% boys) were included. Daytime urinary incontinence (*P* = 0.039) and enuresis (*P* < 0.001) were more diagnosed in primary healthcare, whereas constipation (*P* < 0.001) and abdominal pain (*P* = 0.009) increased from primary to tertiary healthcare. All parent-reported symptoms occurred more frequently than indicated by the physicians. Poor agreement between physicians’ diagnoses and parent-reported symptoms was found (k = 0.16). Locomotor problems prevailed in all healthcare settings, motor skills (*P* = 0.041) and core stability (*P* = 0.015) significantly more in tertiary healthcare.

*Conclusions*: Constipation and abdominal pain (physicians’ diagnoses) and the parent-reported symptoms hard stools and bloating increased from primary to tertiary healthcare. Discrepancies exist between the prevalence’s of physicians’ diagnoses and parent-reported symptoms. Locomotor problems predominate in all healthcare settings.

**Table:** 

**What is Known:** *• Childhood bladder and/or bowel dysfunctions (CCBD) are common.* *• Particularly tertiary healthcare characteristics of CBBD are available*
**What is New:** *• Characteristics of CBBD referred to pelvic physiotherapy are comparable in primary*, *secondary*, *and tertiary healthcare settings.* *• Concomitant CBBD appeared to be more prevalent than earlier reported.* *• Discrepancies exist between referring physicians’ diagnoses and parent-reported symptoms.*

## Introduction

Childhood bladder and/or bowel dysfunctions (CBBD) are common worldwide [1–5]. Bladder dysfunctions are daytime urinary incontinence (DUI), enuresis, and overactive bladder syndrome. The prevalence of DUI and enuresis decreases with age. The peak prevalence of DUI in girls is 8.4% at the age of 7 years, gradually decreasing to 4% in adolescence, whereas the corresponding prevalence rates for boys are 1.4 and 0.9%, respectively [6–8]. Enuresis is a complex condition, involving multiple pathogenic factors [9]. Prevalence’s vary, depending on the definition, approximately 10–20% of all 5-year-olds regularly wet their beds and the prevalence decreases by about 15% each year [10]. Generally accepted is that enuresis is more common in boys than in girls but only until the teenage years [11, 12]. The overactive bladder syndrome is found in 60 to 70% of children with urinary incontinence [13]. Bowel dysfunctions constitute of constipation and fecal incontinence (FI). Estimates of constipation in the general pediatric population range from 0.3 to 8%, with boys and girls equally affected. FI is one of the most common presentations of constipation and is found in up to 84% of constipated children at presentation [4]. FI is estimated to affect 0.8 to 7.8% children in Western societies with reported boys to girls’ ratio’s ranging from 3:1 to 6:1 [14–17]. CBBD have a major impact on a child’s psychosocial functioning. Comorbid behavioral disorders in about 20 to 40% of children with CBBD affect the everyday life of the children and their family [13, 14, 18–21]. The first treatment option of CBBD is a relaxed toileting posture and effective straining to defecate, which requires sufficient locomotor skills [4, 22]. The pelvic floor muscles (PFM) assist in maintaining urinary and fecal continence and, opposite to it, in adequate urination and defecation. Moreover, the PFM cooperate in close synergy with the diaphragm and the abdominal muscles. Therefore, the PFM are also involved in breathing and stabilizing connecting joints and the lower back [23–27]. This means that an unstable or tensed posturing on the toilet, in which the PFM are unable to relax properly, can cause an inadequate urinary flow or bowel movement. From this point of view, CBBD might be related to impaired locomotor skills, although evidence for this hypothesis is lacking.

In the Netherlands, standard medical care for CBBD is initially delivered by the general practitioner, but patients are also allowed to visit a private pelvic physiotherapist (PPT) without referral from a general practitioner or medical specialist. In case of unsatisfactory results, the child can be referred to a pediatrician at a general hospital (secondary healthcare). University-hospital care (tertiary healthcare) is required when secondary healthcare fails. Next, to support SMC, patients can be referred from medical doctors (primary, secondary, and tertiary healthcare) to primary healthcare PPT. Consequently, children visiting a PPT form a heterogeneous group of all ages and CBBD problems.

Limited data is available on the clinical characteristics and complexity of (concomitant) CBBD (physician and parent-reported) in primary and secondary healthcare, while most CBBD studies conducted in tertiary healthcare settings especially focus on treatment effects [6, 28, 29]. The lack of knowledge of patient characteristics, severity of symptoms and co-morbidities throughout healthcare settings may hamper targeting effective treatments. Furthermore, discrepancies are described between physicians’ diagnoses and parent-reported daily symptoms regarding CBBD [30, 31].

The aims of this pragmatic study are to describe (i) the clinical characteristics of CBBD in, and between, primary, secondary and tertiary healthcare settings, (ii) the level of agreement between referring physicians’ diagnoses and questionnaire-based symptoms, reported by parents and (iii) the relation between CBBD and locomotor problems. It was hypothesized that the prevalence’s of CBBD, comorbidities, and locomotor problems would increase, going from lower-to-higher-level healthcare settings.

## Methods

### Study design and population

We performed a cross-sectional study in a sample of children, aged 1–16 years, affected with varying forms of bladder and/or bowel dysfunctions, irrespective of the cause or presence of comorbidity and/or behavioral problems. Except age (1–16 years), no exclusions were made. Children from across the Netherlands and visiting primary healthcare PPT-practices were enrolled. They came on their own initiative (self-initiated visit; primary healthcare) or were referred by either the general practitioner (primary), district hospitals (secondary), or university hospitals (tertiary healthcare settings). Participating PPT’s are all expert pelvic physiotherapists who had completed a professional master’s degree in PPT. Physicians’ diagnoses were established based on patient history and additional assessments (e.g., physical examination, flowmetry, etc.), as documented in accepted pediatric Dutch guidelines [32–35]. Prior to the first visit at the PPT, the parents reported symptoms by completing the Childhood Bladder and Bowel Questionnaire (CBBDQ). Data were retrieved from the electronic patient records of the children.

### Ethics statement

Informed consent was obtained from all parents and children, aged 12 years and older included in the study.

### Web-based electronic patient records

Prior to the first visit at the PPT-practice, parents completed the electronic patient records at home. These included the following components:

#### Patient history

Age, sex, physicians’ diagnoses (possible diagnoses listed with check boxes and an “other options”-text box), chronicity of the CBBD, medication use, comorbidities, and family history. The parent-reported *Strength and Difficulties Questionnaire (*SDQ), a brief validated screening questionnaire, for children age 4–17 was used to assess emotional and behavioral problems in child’s daily life [36–38].

#### Childhood Bladder and Bowel Dysfunction Questionnaire

The CBBDQ is a recently developed evaluative symptom questionnaire based on International Children’s Continence Society recommendations and Rome III criteria for functional gastrointestinal disorders [1, 4, 39, 40], with excellent content and construct validity [40, 41]. The CBBDQ consists of two subscales: (1) the bladder symptoms scale (10 items) and (2) the bowel symptoms scale, including abdominal pain and bloated belly (8 items). The parents were asked to indicate the presence of the symptoms, using a five-point Likert scale [0 (never) to 4 (almost daily or daily)].

#### Locomotor problems

A seven items questionnaire was developed by experts (PPT’s and pediatric physiotherapists) [42] and used as a measure to report problems in locomotor control and motor learning, motor skills (ability to learn to tie shoelaces, cycle or swim) and starting and performing a task, motor control (core stability), and musculoskeletal problems.

### Data analyses

For the descriptive analyses, data are expressed as means and standard deviations for continuous variables or as frequencies and percentages for categorical variables. Comparisons between healthcare settings and gender are made, using analysis of variance for continuous variables and the *χ* ^2^ test for categorical variables.

The SDQ items are coded as “not true”, “somewhat true”, and “certainly true”. The total difficulties score ranges from 0 to 40. Two categories are distinguished: “close to average to slightly raised” (0–16) and “high to very high” (17–40).

To examine the symptom prevalence rates of the CBBDQ, the outcomes were dichotomized, with “never or once in the preceding month” recorded as non-symptomatic and “more than once to (almost) every day in the preceding month” as symptomatic. Possible missing items were imputed as “non-symptomatic”.

To compare in individual children for their referring physicians’ diagnoses versus the parent-reported symptoms (as determined by the CBBDQ), the Cohen’s kappa coefficient is calculated with regard to the categories as follows: “≥1 no BBD”, “≥1 bladder dysfunction”, “≥1 bowel dysfunction”, and “≥1 concomitant CBBD”. A kappa coefficient of 0 to 0.4 is interpreted as poor agreement, 0.41 to 0.75 as “fair to good agreement” and above 0.75 as “excellent agreement” [43].

A *P*-value <0.05 was considered to indicate statistical significance. Statistical analyses were performed with SPSS software, version 23 (IBM Corporation, Somers, NY, USA).

## Results

### Baseline patient characteristics

#### Participants

Table 1 presents the baseline characteristics of the 1748 children (855 boys; mean age 7.6 years [SD 2.8], 893 girls; mean age 7.7 years [SD 2.9]) included from May 2010 to May 2015. No significant differences were found in age and gender between the healthcare settings. One thousand five hundred children (87%) were referred to PPT by a general practitioner or a medical specialist, like a pediatrician, urologist, nephrologist, or pediatric gastroenterologist, while 13% were self-initiated visits.

**Table 1:** Baseline patient characteristics between healthcare settings

		Total	1-HC	2-HC	3-HC	*P* value^a^
		*n* = 1748	*n* = 731	*n* = 906	*n* = 111	
			(41.7)	(51.9)	(6.4)	
Participants						
Age (years)	Mean ± SD	7.7 ± 2.9	7.5 ± 2.9	7.8 ± 2.8	7.9 ± 3.2	0.27
Boys		855 (48.9)	385 (52.6)	414 (45.4)	56 (51.3)	
Age (years)	Mean ± SD	7.6 ± 2.8	7.4 ± 2.8	7.9 ± 2.8	7.6 ± 2.9	0.25
Girls		893 (51.1)	345 (47.3)	493 (53.6)	55 (48.7)	
Age (years)	Mean ± SD	7.7 ± 2.9	7.6 ± 3.0	7.8 ± 2.8	8.2 ± 3.5	0.27
Parents^b^	Single parent	113 (6.7)	46 (6.7)	59 (6.7)	8 (7.1).	
	Two parents	1429 (85.1)	589 (85.7)	749 (84.8)	91 (83.5)	
	Newly formed family	137 (8.2)	52 (7.6)	75 (8.5)	10 (7.3)	
Siblings		1481 (88.2)	603 (87.8)	779 (88.2)	99 (90.8)	0.39
Problems at home^c^		236 (13.5)	96 (13.2)	128 (14.1)	12 (5.1)	0.27
Childhood bladder and bowel dysfunctions (physicians’ diagnoses)						
Daytime urinary incontinence		602 (34.3)	274 (37.6)	296 (32.6)	32 (28.3)	0.039*
Constipation		549 (31.4)	164 (22.5)	341 (37.6)	44 (38.9)	<0.001*
Enuresis		493 (28.2)	243 (33.3)	229 (25.2)	21 (18.9)	<0.001*
Fecal incontinence		362 (20.7)	159 (21.8)	178 (19.6)	25 (22.1)	0.51
Increased voiding frequency		267 (15.3)	114 (15.7)	141 (15.5)	12 (10.6)	0.37
Urinary tract infections		210 (12.0)	48 (6.6)	142 (15.7)	20 (17.7)	<0.001*
Abdominal pain		457 (26.1)	163 (22.4)	259 (28.6)	35 (31.0)	0.009*
Withholding behavior						
At least one bladder dysfunction		1122 (64.2)	486 (66.8)	572 (63.1)	64 (55.6)	0.069
At least one bowel dysfunction		793 (45.4)	286 (36.1)	445 (49.1)	62 (54.9)	<0.001*
CBBD		321 (18.4)	116 (15.9)	183 (20.2)	22 (19.5)	0.083
Other health problems^d^		126 (7.4)	26 (3.7)	80 (8.8)	20 (18.7)	<0.001*
Medication^e^	Bladder	183 (10.5)	77 (10.6)	89 (9.8)	17 (15.0)	0.23
	Bowel	787 (45.4)	287 (39.7)	440 (48.9)	60 (53.1)	<0.001*
Parent-completed SDQ, age 4–16 years						
Analyzed = 1559	Range					
close to average to slightly raised	0–16	1386 (88.9)	547 (87.1)	749 (90.0)	90 (90.9)	
high to very high	17–40	173 (11.1)	81 (12.9)	83(10.0)	9 (9.1)	
Total difficulties score	0–40	9.2 ± 5.7	9.5 ± 5.8	9.0 ± 5.7	8.5 ± 5.5	0.15

*HC* healthcare setting (*1* primary; *2* secondary; *3* tertiary); *SDQ* Strength and Difficulties Questionnaire; *CBBD* Both, bladder and bowel dysfunction. Data are presented as number (*n*) and percentage (%) (or otherwise circumscribed, mean and ± standard deviation (SD))

^a^ANOVA or Pearson’s chi-square

^b^missing 4.9% (*P* = 0.93)

^c^Divorce of parents, death of a parent or grandparent, sibling or pet, moving house, stress at home

^d^Pulmopathy/asthma (2.2%); feeling sick/failure to thrive (1.7%); allergy/eczema (0.8%); neurological disorders (0.7%); endocrine or metabolic disorders (0.5%); musculoskeletal disorders (0.5%); gynecologic disorders (0.4%); complex disorders (0.3%); otopathy (0.2%); cardiopathy (0.2%); psychiatric disorders (0.2%); cystic fibrosis (0.1%); other gastrointestinal tract disorders (0.1%); not otherwise specified (0.4%)

^e^Bladder: overactive bladder, enuresis and current urinary tract infection; bowel: laxatives

*Significant at the P < 0.05 level

#### Referring physicians’ diagnoses

The most common referred diagnoses were daytime urinary incontinence (DUI; 34.3%), constipation (31.4%), enuresis (28.2%), and fecal incontinence (FI; 20.7%) while 26.1% of the children were referred with abdominal pain and 12.0% with urinary tract infections. No differences between health care settings were found with respect to chronicity of any complaints. Laxative use increased going from primary to tertiary care, and these agents were prescribed to 45.4% of the children, whereas medication for bladder dysfunctions was prescribed to 10.5% of the children. A total of 1122 (64.2%) of the children were referred with at least one bladder dysfunction, 793 (45.4%) with at least one bowel dysfunction, and 321 children (18.4%) with both, bladder, and bowel dysfunctions.

Significantly, more DUI (*P* = 0.039) and enuresis (*P* < 0.001) were diagnosed in primary healthcare. Urinary tract infection, especially among girls, increased going from primary to tertiary healthcare settings (P = <0.001). Boys had less frequent bladder dysfunctions (*P* = 0.022) in tertiary care and less bowel dysfunctions in primary healthcare (*P* < 0.001). Whereas constipation (*P* < 0.001) and abdominal pain (*P* = 0.009) were more diagnosed in secondary and tertiary healthcare and more prevalent among girls (*P* < 0.001) (Table 2). FI was more common among boys in all settings (*P* < 0.001). The number of children referred with “at least one bowel dysfunction” increased, going from primary to tertiary healthcare settings (*P* < 0.001). Other health problems, such as lung diseases, also increased significantly from 3.7 to 18.7% (*P* < 0.001).

**Table 2:** Gender-specific childhood bladder and bowel dysfunctions between healthcare setting (physicians’ diagnosis)

	Gender	Total	1-HC	2-HC	3-HC	*P* value^a^
		*n* = 1748	*n* = 731	*n* = 906	*n* = 111	
			(41.8)	(51.9)	(6.4)	
Childhood Bladder and Bowel Dysfunctions (physicians’ diagnoses)						
Daytime urinary incontinence	B versus G^b^					0.65
	boys	229 (35.0)	138 (36.0)	147 (35.5)	14 (24.1)	0.20
	girls	303 (33.9)	136 (39.4)	149 (30.2)	18 (32.7)	0.022*
Constipation	B versus G^b^					<0.001*
	boys	225 (26.3)	56 (14.5)	149 (36.0)	20 (35.7)	<0.001*
	girls	324 (36.6)	108 (31.3)	192 (38.9)	24 (43.6)	0.039*
Enuresis	B versus G^b^					<0.001*
	boys	320 (37.4)	160 (41.6)	147 (35.5)	13 (23.2)	0.016*
	girls	173 (19.4)	83 (24.1)	82 (16.6)	8 (14.5)	0.018*
Fecal incontinence	B versus G^b^					<0.001*
	boys	232 (27.1)	104 (27.0)	113 (27.3)	15 (26.8)	0.048*
	girls	130 (14.6)	56 (16.2)	65 (13.2)	9 (16.4)	0.22
Increased voiding frequency	B versus G^b^					0.51
	boys	136 (15.9)	56 (14.6)	74 (17.9)	6 (10.3)	0.23
	girls	131 (14.7)	58 (16.8)	67 (13.6)	6 (10.9)	0.32
Urinary tract infections	B versus G^b^					<0.001*
	boys	18 (2.1)	6 (1.6)	11 (2.7)	1 (1.7)	0.63
	girls	192 (21.5)	42(12.2)	131(26.6)	19 (34.5)	<0.001*
Abdominal pain	B versus G^b^					<0.001*
	boys	167 (19.5)	61 (15.9)	93 (22.5)	13 (22.4)	0.056
	girls	290 (32.5)	102 (29.6)	166 (33.7)	22 (40.0)	0.22
Withholding behavior	B versus G^b^					0.71
	boys	234 (27.4)	121 (31.4)	102 (24.6)	11 (19.6)	0.040*
	girls	252 (28.2)	124 (35.9)	110 (22.3)	18 (32.7)	<0.001*
At least one bladder dysfunction	B versus G^b^					0.32
	boys	554 (64.8)	265 (68.8)	260 (62.8)	29 (51.8)	0.022*
	girls	568 (63.6)	222 (64.3)	312 (63.3)	34 (61.8)	0.91
At least one bowel dysfunction	B versus G^b^					1.0
	boys	388 (45.4)	142 (36.9)	214 (51.7)	32 (56.9)	<0.001*
	girls	405 (45.4)	145 (42.0)	231 (46.9)	29 (52.7)	0.20
Both, bladder and bowel dysfunction	B versus G^b^					0.76
	boys	160 (18.7)	63 (16.4)	88 (21.3)	9 (16.1)	0.18
	girls	161 (18.0)	54 (15.7)	95 (19.3)	12 (21.8)	0.30

*HC* healthcare setting (*1* primary; *2* secondary; *3* tertiary). Data are presented as number (*n*) and percentage (%)

^a^Pearson’s chi-square or Fisher’s exact test

^b^B versus G; boys versus girls between healthcare settings

*Significant at the *P* < 0.05 level

#### Strength and Difficulties Questionnaire

As depicted in Table 1, no significant differences in SDQ were found between healthcare settings. On a range of 0–40, the mean total SDQ difficulties scores were 9.5 (SD 5.8), 9.0 (SD 5.7), and 8.5 (SD 5.5) for primary, secondary, and tertiary healthcare, respectively, and 11.1% of the children had a total SDQ-difficulties score over 16, indicating the children had emotional or behavioral problems.

### Parent-reported symptoms

Parent-reported symptoms are described in Table 3 (proportions between healthcare settings) and Table 4 (gender-specific). Based on the parent-reported symptoms, bladder problems like DUI occurred more often in primary healthcare than in secondary and tertiary healthcare (*P* = 0.023). High prevalence of ignoring the urge to urinate (53.0%) and urgency (56.6%) were found in all healthcare settings, whereby “ignoring” decreased significantly (*P* = 0.023) from primary to tertiary healthcare setting. Boys have more DUI than girls (*P* = 0.006), especially in secondary and tertiary healthcare settings and post-micturition dribble (*P* = 0.003), decreasing from primary to tertiary healthcare settings. Boys were more likely to suffer from enuresis than girls (*P* < 0.001). In contrast, girls wake up at night to urinate more often (*P* = 0.045).

**Table 3:** Childhood bladder and bowel dysfunctions (parent-reported) between HC-settings

	Total	1-HC	2-HC	3-HC	*P* value^a^
	*n* = 1748	*n* = 730	*n* = 907	*n* = 111	
Items of the Childhood Bladder and Bowel Dysfunction Questionnaire^b^					
1 Passes urine more than 8 times during the day	676 (38.7)	289 (39.6)	355 (39.1)	32 (28.8)	0.087
2 Wets underwear and /or outer clothing during the day	859 (49.1)	383 (52.5)	430 (47.4)	46 (41.4)	0.031*
3 Loses some drops of urine immediately after urinating has finished	684 (39.1)	302 (41.4)	347 (38.3)	35 (31.5)	0.40
4 Loses urine within the hour after urinating has finished	518 (29.6)	225 (30.8)	267 (29.4)	26 (23.4)	0.28
5 Seems to ignore the urge to urinate	927 (53.0)	407 (55.8)	473 (52.1)	47 (42.3)	0.023*
6 Uses tricks to stay dry, like wriggling or forcefully crossing the legs	597 (34.2)	261 (35.8)	303 (33.4)	33 (29.7)	0.36
7 Experiences a sudden uncontrollable urge to urinate	989 (56.6)	422 (57.8)	510 (56.2)	57 (51.4)	0.42
8 Postpones first urination in the morning	505 (28.9)	203 (27.8)	268 (29.5)	34 (30.6)	0.68
9 Wets the bed or diaper during sleeping periods	742 (42.4)	339 (46.4)	362 (39.9)	41 (36.9)	0.014*
10 Wakes up at night to urinate	353 (20.2)	128 (17.5)	203 (22.4)	22 (19.8)	0.052
11 Has two or fewer bowel movements per week	373 (21.4)	169 (23.2)	176 (19.4)	28 (25.2)	0.11
12 Stains or soils the underwear with stools	778 (44.5)	335 (45.9)	385 (42.4)	58 (52.3)	0.09*
13 Has hard stools or painful bowel movements	414 (23.7)	150 (20.5)	232 (25.6)	32 (28.8)	0.025*
14 Has large amounts of stool (that may obstruct the toilet)	345 (19.7)	147 (20.1)	168 (18.5)	30 (27.0)	0.10
15 Postpones bowel movements	616 (35.2)	292 (40.0)	286 (31.5)	38 (34.2)	0.002*
16 Experiences a sudden uncontrollable urge to defecate	763 (43.7)	326 (44.7)	389 (42.9)	48 (43.6)	0.77
17 Has abdominal pain	699 (40.1)	270 (37.1)	383 (42.3)	46 (41.4)	0.10
18 Has a bloated belly	415 (23.7)	157 (21.5)	214 (23.6)	44 (39.6)	<0.001*
At least one bladder symptom	1566 (89.6)	668 (91.5)	805 (88.8)	93 (83.8)	0.023*
At least one bowel symptom	1404 (80.3)	589 (80.7)	722 (79.6)	93 (83.8)	0.55
Combined bladder and bowel symptom	1266 (72.4)	539 (73.8)	647 (71.3)	80 (72.1)	0.53

*HC* healthcare setting (*1* primary, *2* secondary, *3* tertiary). Data are presented as number (*n*) and percentage (%)

^a^Pearson’s chi-square

^b^Likert scale for symptoms on all items are (never–(nearly) every day) dichotomized: “Never or once in the past month” classified as “non-symptomatic” (no); “more than once in the past month to (nearly) every day in the past month” classified as “symptomatic” (yes). Missing items were imputed as “non-symptomatic”

*Significant at the *P* < 0 05 level

**Table 4:** Childhood Bladder and Bowel Dysfunction Questionnaire (parent-reported symptoms) gender-specific

	Gender	Total	1-HC	2-HC	3-HC	P value^a^
		*n* = 1748	*n* = 730	*n* = 907	*n* = 111	
Items of the Childhood Bladder and Bowel Dysfunction Questionnaire^b^						
1 Passes urine more than 8 times during the day	B versus G^c^					0.057
	boys	350 (40.9)	163 (42.3)	170 (41.1)	17 (30.4)	0.23
	girls	326 (36.5)	126 (36.5)	185 (37.5)	15 (27.3)	0.33
2 Wets underwear and /or outer clothing during the day	B versus G^c^					0.006*
	boys	449 (52.2)	213 (47.4)	212 (48.8)	32 (57.1)	0.17
	girls	410 (45.9)	170 (49.3)	218 (44.2)	22 (40.0)	0.23
3 Loses some drops of urine immediately after urinating finished	B versus G^c^					0.003*
	boys	490 (57.3)	173 (44.9)	173 (41.8)	19 (33.9)	0.26
	girls	319 (35.7)	129 (37.4)	174 (35.3)	16 (29.1)	0.47
4 Loses urine within the hour after urinating has finished	B versus G^c^					0.08
	boys	270 (31.6)	122 (31.7)	131 (31.6)	17 (30.4)	0.98
	girls	248 (27.8)	103 (29.9)	136 (27.6)	9 (16.4)	0.12
5 Seems to ignore the urge to urinate	B versus G^c^					0.006*
	boys	482 (56.4)	227 (59.0)	234 (56.5)	21 (37.5)	0.010*
	girls	445 (49.8)	180 (52.2)	239 (48.5)	26 (47.3)	0.53
6 Uses tricks to stay dry, like wriggling or forcefully crossing legs	B versus G^c^					0.76
	boys	295 (34.5)	147 (38.2)	136 (32.9)	12 (21.4)	0.030*
	girls	302 (33.8)	114 (33.0)	167 (33.9)	21 (38.2)	0.76
7 Experiences a sudden uncontrollable urge to urinate	B versus G^c^					0.04*
	boys	505 (59.1)	232 (60.3)	244 (58.9)	27 (48.2)	0.48
	girls	484 (54.2)	190 (55.1)	266 (54.0)	28 (50.9)	0.84
8 Postpones first urination in the morning	B versus G^c^					0.09
	boys	231 (27.0)	105 (27.3)	112 (27.1)	14 (25.0)	0.94
	girls	274 (30.7)	98 (28.4)	156 (31.6)	20 (36.4)	0.39
9 Wets the bed or diaper during sleeping periods	B versus G^c^					<0.001*
	boys	445 (52.0)	210 (54.5)	208 (46.7)	29 (48.2)	0.40
	girls	297 (33.3)	129 (37.4)	154 (31.2)	14 (25.5)	0.079
10 Wakes up at night to urinate	B versus G^c^					0.01*
	boys	151 (17.7)	56 (14.5)	87 (21.0)	8 (14.3)	0.045*
	girls	202 (22.6)	72 (20.9)	116 (23.5)	14 (25.5)	0.58
11 Has two or fewer bowel movements per week	B versus G^c^					0.44
	boys	176 (20.6)	77 (20.0)	85 (20.5)	14 (25.0)	0.014*
	girls	197 (22.1)	92 (26.7)	91 (18.5)	14 (25.5)	0.69
12 Stains or soils the underwear with stools	B versus G^c^					0.001*
	boys	440 (51.5)	183 (47.5)	202 (48.8)	30 (53.6)	0.69
	girls	363 (40.6)	152 (44.1)	183 (37.1)	28 (50.9)	0.037*
13 Has hard stools or painful bowel movements	B versus G^c^					<0.001*
	boys	164 (19.2)	58 (15.1)	91 (22.0)	15 (26.8)	0.015*
	girls	250 (28.0)	92 (26.7)	141 (28.6)	17 (30.9)	0.73
14 Has large amounts of stool (that may obstruct the toilet)	B versus G^c^					0.57
	boys	164 (19.2)	65 (16.9)	84 (20.3)	15 (26.8)	0.16
	girls	181 (20.3)	82 (23.8)	84 (17.0)	15 (23.7)	0.024*
15 Postpones bowel movements	B versus G^c^					<0.001*
	boys	337 (39.4)	172 (44.7)	150 (36.2)	15 (26.8)	0.021*
	girls	279 (31.2)	120 (34.8)	136 (27.6)	23 (41.8)	0.002*
16 Experiences a sudden uncontrollable urge to defecate	B versus G^c^					0.06
	boys	402 (47.0)	187 (48.6)	192 (46.4)	23 (41.1)	0.54
	girls	361 (40.5)	139 (40.3)	197 (40.0)	25 (46.3)	0.66
17 Has abdominal pain	B versus G^c^					<0.001*
	boys	299 (35.0)	128 (33.3)	150(36.2)	21 (37.5)	0.22
	girls	400 (44.9)	142 (41.3)	233 (47.5)	25 (45.5)	0.64
18 Has a bloated belly	B versus G^c^					<0.001*
	boys	162 (18.9)	67 (17.1)	77 (18.6)	18 (32.1)	0.030*
	girls	253 (28.3)	90 (26.1)	137 (27.8)	26 (47.3)	0.005*
At least one bladder symptom	B versus G^c^					0.27
	boys	773 (90.4)	355 (92.2)	371 (89.6)	47 (83.9)	0.11
	girls	793 (88.8)	313 (90.7)	493 (88.0)	46 (83.6)	0.22
At least one bowel symptom	B versus G^c^					0.93
	boys	686 (80.2)	312 (81.0)	331 (80.0)	43 (76.8)	0.74
	girls	718 (80.4)	227 (80.3)	391 (79.3)	50 (90.9)	0.12
Combined bladder and bowel symptom	B versus G^c^					0.61
	boys	624 (73.0)	286 (74.3)	300 (72.5)	38 (67.9)	0.57
	girls	642 (71.9)	253 (73.3)	347 (70.4)	42 (76.4)	0.48

*HC* healthcare setting (1 primary, *2* secondary, *3* tertiary). Data are presented as number (*n*) and percentage (%)

^a^Pearson’s chi-square

^b^Likert scale for symptoms (never–(nearly) every day) dichotomized: “Never or once in the past month” classified as “non-symptomatic” (no); “more than once in the past month to (nearly) every day in the past month” classified as “symptomatic” (yes). Missing items were imputed as “non-symptomatic”

^c^B versus G; boys versus girls between healthcare settings

*Significant at the *P* < 0.05 level

### Locomotor problems

Locomotor problems prevailed in all healthcare settings and the prevalence increased with higher-level healthcare settings (Table 5). Parents indicated that 14.5% of all children have had problems in motor learning, 9.2% had problems in core stability, and 10.8% had an increased muscle tension. Children referred from tertiary healthcare settings experienced more problems than those from primary and secondary settings. Significant differences between healthcare settings were found in motor skills (*P* = 0.041) and core stability problems (*P* = 0.015). No differences were found with respect to having problems starting or performing a task or musculoskeletal problems.

**Table 5:** Locomotor problems (parent-reported)

	Total	1-HC	2-HC	3-HC	*P* value^a^
	*n* = 1674	*n* = 684	*n* = 881	*n* = 109	
Motor learning					
Reduced manual dexterity^b^	175 (10.5)	73 (10.7)	88 (10.0)	14 (12.8)	0.64
Problems of motor skills^c^	286 (17.1)	99 (14.6)	164 (19.0)	23 (21.3)	0.041*
Problems starting and performing a task^c^	266 (15.9)	101 (14.8)	145 (16.5)	20 (18.5)	0.50
Motor control					
Problems in core stability^c^	154 (9.2)	51 (7.5)	86 (9.8)	17 (15.7)	0.015*
Musculoskeletal					
Increased muscle tension	180 (10.8)	76 (12.1)	94 (11.3)	10 (10.1)	0.83
Reduced muscle tension	28 (1.7)	13 (1.8)	13 (1.4)	2 (1.8)	0.91

*HC* healthcare setting (*1* primary, *2* secondary, *3* tertiary). Data are presented as number (*n*) and percentage (%)

^a^Pearson’s chi-square

^b^Coding of dummy variables: Manual dexterity: normal/increased = 0; reduced = 1

^c^Coding of dummy variables: Locomotor regarding stability and skills (such as tying shoelaces, swimming), starting or performing a task never/sometimes = 0; often = 1

*Significant at the *P* < 0.05 level

## Discussion

To our knowledge, this cross-sectional study is the first to describe clinical characteristics of (concomitant) CBBD in different healthcare settings, in a large sample of 1748 affected children, visiting primary PPT-practices. In this pragmatic study, all referred children, irrespective of age, complexity of complaints, or comorbidities were included, yielding a heterogeneous cohort reflecting routinely PPT-practice. Although we have hypothesized that the prevalence’s of CBBD, comorbidities, and locomotor problems would increase going from lower-to-higher-level healthcare settings, the results of our study could only be confirmed for the physicians’ diagnoses constipation and abdominal pain and the parent-reported symptoms hard stools, abdominal pain, bloating, problems in core stability, and the existence of other health problems. In contrast, DUI, ignoring the urge to urinate and enuresis decreased going from lower-to-higher-level healthcare settings. Poor agreement exists between referred physicians’ diagnoses and questionnaire-based parent-reported symptoms. Locomotor problems prevailed in all healthcare settings. Children referred from tertiary healthcare settings experienced more problems in motor skills and core stability than those from primary and secondary settings.

Some striking gender differences appeared when comparing our results with earlier studies. Significantly, more girls were suffering from constipation, abdominal pain, and urinary tract infection (physician’ diagnosis), whereas boys experienced more DUI, hard stools or painful bowel movements (parent-reported symptoms) and fecal incontinence and enuresis (physician’s diagnosis and parent-reported symptoms). Overall, estimates of presented prevalence figures differ greatly and depend not only on the clinical setting, but also on the heterogeneity of the criteria used for defining or diagnosing bladder or bowel dysfunctions. Standardized use of the accepted International Children’s Continence Society recommendations and/or the Rome III criteria for functional gastrointestinal disorders would facilitate study comparability.

Concomitant CBBD was equally distributed over all three healthcare settings and diagnosed by physicians in 18.4% of the children compared to 72.4% when considering parent-reported symptoms. In fact, all parent-reported symptoms occurred more frequently than indicated by the physicians’ diagnoses, especially when considering combined bladder and bowel symptoms. This discrepancy between physicians’ diagnoses and CBBDQ outcomes may due to both, physicians and parents. When physicians focus on questioning for bladder symptoms or bowel symptoms, then concomitant CBBD will be missed. Next, is the lack of parent’s knowledge of linking complaints of CBBD. Besides, filling in questionnaires raise the attention to certain symptoms. Therefore, when a physician does not explicitly ask for all CBBD symptoms, the parents or children most likely will not report them. Therefore, caregivers should be made aware of this discrepancy, to prevent the risk of inadequately diagnosing CBBD, to promote favorable therapy outcomes, and to reduce the risk of relapses. Using a CBBD questionnaire might facilitate elicitation of all relevant symptoms.

Locomotor problems prevailed in all healthcare settings. Epidemiological studies have shown that about 6% of all school-age children are described by experts and parents as uncoordinated in their fine and gross motor skills [44]. In our study, parents indicated that 14.5% of all children have had problems in motor learning and 9.2% had problems in core stability. This finding could not be explained by concomitant physical problems or comorbidities, as the number of these children was low and did not differ significantly between healthcare settings. Dysfunction of the pelvic floor muscles (PFM) and cooperating abdominal muscles is an integral component of the pathophysiology of CBBD [45–47]. Only Chase et al. [48] have examined whether different trunk musculoskeletal characteristics might be related to defecation difficulties. In agreement with our results, they found rather high prevalence rates for core stability and motor skills problems in children, supporting the hypothesis, that locomotor problems exists, indicating that dysfunctions of the muscles may be associated with CBBD [23–25, 27, 49–51]. Hence, pelvic physiotherapists, as musculoskeletal specialists, might play a role in treating children with CBBD [45, 48, 52–55].

Studies have reported that children with CBBD are at increased risk of psychosocial, behavioral, or psychological disorders [13, 56]. Although emotional or behavioral problems were present in 11.1% of the children, no association between behavioral problems and CBBD could be confirmed in all three healthcare settings. The SDQ scores did not deviate far from the norm scores reported in the literature, indicating that our sample appeared to be representative of the normal Dutch population of children aged 1–16 years.

Some limitations might affect the interpretation of our findings. First, diagnoses used by physicians were based on heterogeneity of criteria. Secondly, although CBBDQ has been evaluated for validity aspects, further research is required to define its psychometric properties and to justify its use in research and clinical practice. Moreover, symptoms, indicated by the CBBDQ, were not verified by means of diagnostic testing. Then, generalization of our findings may be hampered by the fact that healthcare systems and therewith referral patterns may differ per country. Next, it is unclear if this sample of children is a typical subset of the broader population, and whether the medical doctors have referred all children with functional BBD, or only the children who failed SMC. Finally, data on the locomotor problems were obtained through parental reports and were not confirmed by a questionnaire of adequate psychometric evaluation or by musculoskeletal examination. Further, well-designed studies are necessary to assess whether children with CBBD have more locomotor problems compared to their healthy peers.

Despite the aforementioned limitations, we feel that our study has strong points, such as a large sample that approximates the average patient in all healthcare settings with no restrictions regarding CBBD definition and comorbidities, and our study is one of first taking in account the motor control problems in relation to CBBD.

## Conclusion

The present pragmatic study is one of the first to report the clinical characteristics of children with various forms of CBBD referred to PPT from primary, secondary, and tertiary healthcare settings. The results indicate that our hypothesis could only be confirmed for the physicians’ diagnoses constipation, abdominal pain, the existence of other health problems, and the parent-reported symptoms hard stools, bloating, and problems in core stability. More girls were suffering from constipation, abdominal pain, and urinary tract infections (physicians’ diagnoses), boys from DUI and experiencing hard stools or painful bowel movements (parent-reported symptoms) and fecal incontinence and enuresis (physicians’ diagnoses and parent-reported symptoms). The major discrepancy between physicians’ diagnoses and the symptoms, reported by the parents, raises the question whether parents are aware that their child has concomitant bladder and bowel dysfunctions when visiting a physician. Using a combined CBBD questionnaire might reduce the risk of inadequate diagnosing CBBD. Finally, locomotor problems prevailed in all healthcare settings. Since both the PFM (contributing to urination, defecation, continence, intra-abdominal pressure generation, antigravity support, and lumbo-pelvic stability) and locomotor problems might be a part of CBBD, pelvic physiotherapists can be considered to be involved in the healthcare of children affected with CBBD.

## Abbreviations

CBBD: childhood bladder and bowel dysfunctions
CBBDQ: Childhood Bladder and Bowel Dysfunctions Questionnaire
DUI: Daytime urinary incontinence
FI: Fecal incontinence
ICCS: International Children’s Continence Society
SDQ: Strengths and Difficulties Questionnaire
SMC: Standard medical care
PFM: Pelvic floor muscles
PPT: Pelvic physiotherapy
